# 3D-Printed Electrode/Electrolyte Architectures for High-Performance Lithium-Ion Batteries: Mechanisms, Materials, and Challenges

**DOI:** 10.1021/acsomega.5c02247

**Published:** 2025-07-01

**Authors:** Xiaofei Lou, Li Zhao, Yang Gao, Xiaohui Nan

**Affiliations:** 1 College of Mechatronic Engineering, 12680 North Minzu University, Yinchuan, Ningxia 750021, China; 2 Ningxia Engineering Research Center for Hybrid Manufacturing System, Yinchuan Ningxia 750021, China

## Abstract

The advent of 3D printing technology has a significant development in the field of science and technology. As an important energy storage device, lithium-ion batteries are progressively incorporating 3D printing technology to construct nanomicro structures, thereby enhancing the electrochemical performance. The precise control of the structure and shape of the anode/cathode materials allows for improvements in the energy density and cycling performance of the batteries. Furthermore, 3D printing technology can be employed in the fabrication of solid-state electrolytes for lithium-ion batteries to enhance their conductivity and durability. The application of 3D printing technology in lithium-ion batteries has considerable potential for improving battery performance and simplifying construction processes. Thus, this review has sought to provide a comprehensive overview of the mechanisms underlying the various 3D printing methods while also analyzing the different 3D printing methods adopted in electrode/electrolyte manufacturing over recent years. What’s more, it has discussed the challenges associated with the 3D printing method in manufacturing lithium-ion batteries.

## Graphical abstract

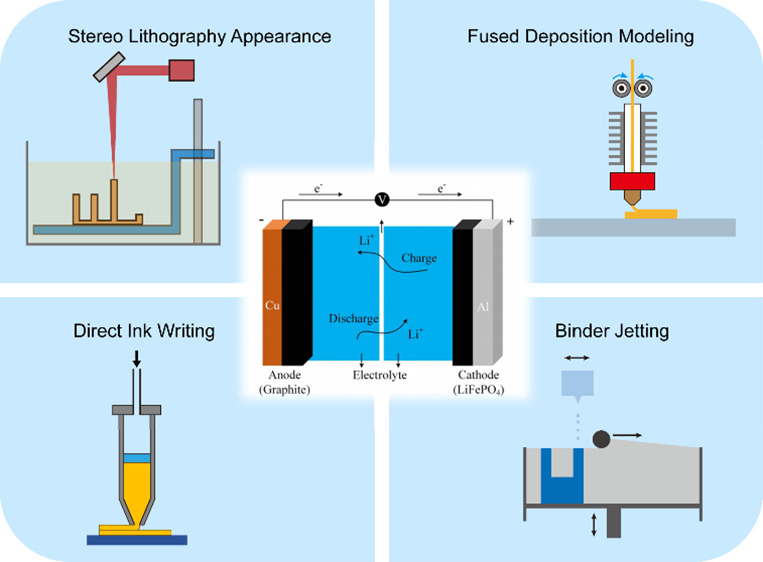

## 1 Introduction

To address the dual challenges of the gradual consumption of fossil fuels and increasing global warming, the development and adoption of green energy have become an urgent priority for humanity. The utilization of energy is depended upon the reserve and transfer of electrical energy, irrespective of whether the source of energy is wind, hydrogen or solar power. Electrochemical energy storage devices are of significant consequence in this regard.^1–5^ Among the various storage devices, lithium-ion batteries are widely used due to their excellent capacity, high operating voltage, superior charge retention capability, strong charge retention ability, wide operating temperature range, long cycle life, and high safety *etc.*.^6,7^ Lithium-ion batteries are composed of anode, cathode, separator, electrolyte, collector, binder and conductive agent *etc.*.^8–10^ The reasonable and effective design of the electrode is a pivotal factor in optimizing the electrochemical properties of lithium-ion batteries.

Conventional electrode preparation process typically employs the use of binder to coat the electrode material on current collector, which is copper for anodes or aluminum for cathodes. The binder and collector as well as conductive additives are both nonelectrochemically active substances but account for a significant portion of the battery, which has a harmful impact on the energy density of lithium-ion batteries.^11^ Furthermore, conventional preparation techniques are constrained in their ability to regulate the geometry and structure of electrodes-electrolytes. In contrast, 3D printing technology offer a means of precise control the structural characteristics of materials, including dimensions, pore distribution and macroscopic shape, which can enhance the energy/power density of lithium-ion batteries.^12^ Compared with traditional 2D structure fabrication techniques, 3D printing can further construct a more efficient and effective way to improve the energy/power density of lithium-ion batteries.^13^ Through advanced and rational electrode structure design, the expressiveness of the electrodes, power density, energy density, cycle life, and mechanical properties of the assembled lithium-ion batteries can be significantly enhanced.^14^ In view of the popularization and application of 3D printing technology in lithium-ion batteries, this paper reviews the principles of common 3D printing mechanism of electrode–electrolyte in detail, followed by an overview of different anode–cathode materials and electrolyte preparation processes. Then, the principles of 3D printing technology in the recent years according to the component of lithium-ion batteries are summarized. Lastly, the unaddressed challenges and problems of 3D printing technology in lithium-ion batteries preparation process are discussed.

## 2 3D Printing Methods

3D printing, as known as additive manufacturing technology, is the process of creating objects by placing layers of material on top of another to form a 3D structures.^15,16^ Based on the manufacturing principles, 3D printing technologies can be categorized as fused deposition molding, direct ink writing, stereo lithography appearance and binder jetting.

### 2.1 Fused Deposition Modeling

Fused deposition modeling (FDM) is a molding technology widely used in the field of 3D printing.^17^ It is a relatively simple method in which a low-melting-point filamentary material (usually thermoplastic, wax, ABS, nylon, *etc.*) is melted into a liquid state and extruded through a nozzle, and then deposited onto the substrate to form a 3D layer-by-layer structure.^18,19^ The material can solidify over time to achieve the multilayered deposition and eventual molding. The key to FDM is controlling the temperature of the molten material and the extrusion speed as well as the movement and positioning of the platform.^20,21^ By accurately controlling these parameters, the 3D printer can deposit the material at the correct position and height of each layer, ultimately forming a 3D object structure with the desired geometry.^22^ The FDM printing method has a simple principle of system construction and operation, low maintenance costs and safe system operation.^23,24^ However, lots of disadvantages exist, for example, the printing speed is slow and the surface of the molded part has obvious stripes, which has reduced the quality of eventual products. In addition, the strength along the vertical direction of the molding axis is weak, lowering down the support of the microstructure which is essential for electrode.^25^

### 2.2 Direct Ink Writing

Direct ink writing (DIW) of viscoelastic materials is an important branch of 3D printing technology.^26^ The key to DIW printing is the use of concentrated solutions of polymers, volatile organics and resins with suitable fluid characteristic. The viscosity of the printing ink drops drastically by shear stress and present a self-supporting properties.^27,28^ These shear-stress yielding fluids can be described by the Herschel-Bulkley model:

$$\tau =\tau_{y}+K\gamma^{n}$$

where τ is the shear stress,τ_*y*_ is the yield stress, *K* is the viscosity parameter, γ is the shear rate, *n* is the flow index. Typical values for the appropriate viscosity of the printing ink is decided by nozzle diameter and printing speed. To direct the flow of the printing material through the nozzle, the stresses applied to the nozzle must exceed the yield stress τ_*y*_ of the printing material. When the material leaves the nozzle, it quickly recovers to its original value τ_*y*_ and shear modulus of elasticity G’.^29^ Additional thermal treatments are often required in DIW printing to allow for complete curing of the printing part.^30^ DIW can print any material as long as the precursor inks can be designed to exhibit the appropriate rheological behavior.^31,32^ As shown in Figure 1, the printing ink is extruded out of the nozzle by applying pressure. The ink is mainly consisted of polymers, ceramics, glass, metals, *etc*. This technology has received huge attention for its ability to handle the widest range of materials in the fabrication of complex and versatile 3D structures.

**Figure 1:**
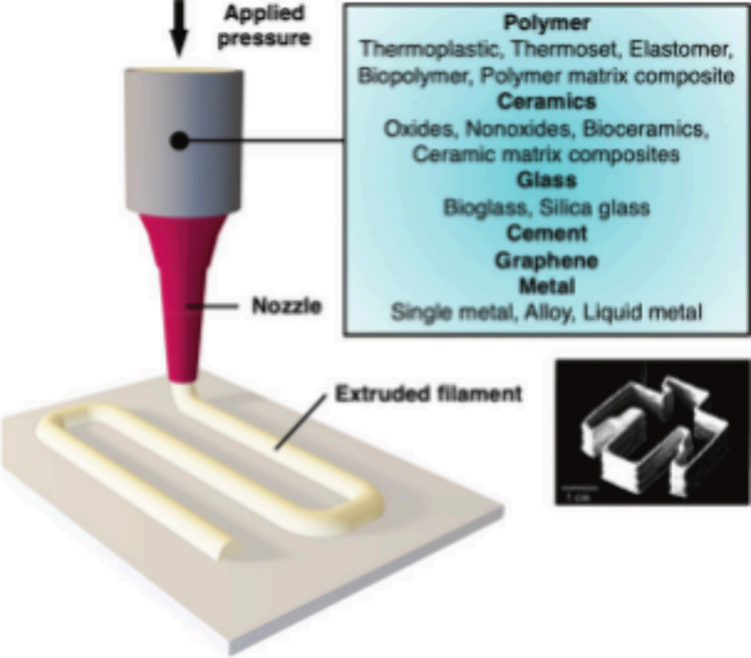
Schematic diagram of the DIW^12^ Reprinted with permission from Saadi, M.; Maguire, A.; Pottackal, N. T.; Thakur, M. S. H.; Ikram, M. M.; Hart, A. J.; Ajayan, P. M.; Rahman, M. M. J. A. M., Direct ink writing: a 3D printing technology for diverse materials. 2022, 34 (28), 2108855. Copyright 2022 John Wiley and Sons.

### 2.3 Stereo Lithography Appearance

Stereo lithography appearance (SLA) is a technique for fabricating micronanostructures for various materials. The fundamental principle of this process is to utilize the interference and diffraction effects of light to transfer the pattern of light onto the photoresist through irradiation of light and a photosensitization reaction of the photoresist. This is followed by the transfer of the pattern onto the underlying substrate, which is achieved through either chemical or physical means, thus enabling the fabrication of designed structures.^33,34^ The photoresist material must be a photosensitive polymer that is capable of developing a chemical reaction when irradiated with ultraviolet (UV) light.^35^ By regulating the characteristics of the light source, mask, and lithographic material, it is possible to achieve the fabrication of micronano structures with high resolution and precision. The advantages of SLA technology include the capacity to create products with complex structures and high precision, as well as the elimination of cutting tools and molds, which significantly reduces the time required for the transition from design to molding.^36^ Nevertheless, SLA technology has its disadvantages, such as the restricted strength, stiffness and heat resistance of the resins employed, as well as the necessity for a more controlled working environment.^37^ SLA is widely utilized in a wide range of fields, including semiconductor fabrication, microelectronic devices, biochips, and micromechanical systems.^38,39^

### 2.4 Binder Jetting

Binder jetting (BJ) technology forms the desire part by jetting the binder onto a bed of plastic powders, which uses the interaction of the material jetting and sintering processes to generate compact components.^40^ The utilization of binder jetting necessitates the employment of bespoke jetting apparatus, including nozzles, sprayers, and other analogous devices. Such devices must be capable of uniformly applying the binder in the form of a jet to the surface of the material to be bonded.^41^ The jetting device is typically responsible for propelling the binder outward through the application of compressed air or other external forces and it allows the use of a diverse range of materials, including metals, ceramics, sand, and others, thereby offering a vast array of possibilities for the fabrication of an extensive variety of products.^42^ The potential applications of binder jetting technology are numerous and diverse, spanning a range of sectors including aerospace, automotive manufacturing, medical, and consumer products.^43,44^

The comparisons of the advantages and limitations of different 3D printing technologies are listed in Table 1.

**Table 1:** Comparative Analysis of the Advantages and Limitations of Common 3D Printing Technologies

3D printing technology	advantages	limitations	examples of applications	refs
**FDM**	low cost, environmentally friendly, high utilization of raw materials	low accuracy, weak mechanical properties	ABS/montmorillonite nanocomposites	^45^
			MWCNT/montmorillonite	^46^
			MoS_2_/carbon/polylactic acid filament	^47^
**DIW**	various shapes, low temperature required, low cost	relatively weak product mechanical properties	LiFePO_4_@MgO	^48^
			Li_4_Ti_5_O_12_, LiNi_0.815_Co_0.15_Al_0.035_O_2_, and LiFePO_4_ half-cells	^49^
			carbon nanofibers/alumina	^50^
			3D-SiC/Al composites	^51^
**SLA**	high quality, high speed, high resolution	high cost, slow speed	PEGDA/nHAP biomaterials	^52^
			POSS/MESO composites	^53^
			CNCs-IAME/IESO composite resin	^54^
**BJ**	multimaterial availability, high precision, high volume production capability	limited product strength, fewer raw material choices	MXene composite	^55^
			diamond/copper composites	^56^

## 3 3D Printing of Electrode Materials for Lithium-Ion Batteries

### 3.1 Anode Materials

#### 3.1.1 Lithium

The earliest anode material for lithium secondary batteries was lithium metal, which has the lowest electrode potential (−3.045 V) and relatively high theoretical mass specific capacity (3860 mAh·g^–1^) of known materials.^57–59^ However, when lithium metal is employed as the anode, the deposition of lithium on the surface of the electrode during the charging and discharging processes is prone to exhibit nonuniformity. This phenomenon, whereby lithium is deposited at an accelerated rate at specific sites, is referred to as the formation of “lithium dendrites”.^60^ Once a certain length of dendrites has been reached, the material will undergo a fracturing process, resulting in the formation of what is known as “dead lithium”.^61^ The “dead lithium” is used to describe a reduction in the quantity of electrode active substance, which in turn affects the electrochemical performance of the battery. Conversely, a reduction in the quantity of electrode active substance will have an adverse effect on the electrochemical performance of the battery. From one perspective, the presence of “dead lithium” will result in a reduction of the active substance content of the electrode, which in turn will have an adverse impact on the electrochemical performance of the battery. From another perspective, the presence of “dead lithium” will give rise to safety concerns, including the potential for short circuits and explosions within the battery.^62,63^ Lithium also exhibits high reactivity, which can result in the depletion of the active substance and the potential for safety hazards when it reacts with the electrolyte.^64^ Furthermore, the low coulombic efficiency and the gradual increase of the negative electrode overpotential result in a constant decay in the capacity of the lithium anode during the cycling process. Consequently, there is a continued need to enhance the commercialization of lithium metal. Moreover, the cycling stability of the lithium anode must be optimized in order to facilitate the widespread adoption of lithium metal anodes.^65,66^ Notwithstanding the considerable difficulties inherent to lithium, its noteworthy characteristics of high capacity and low overpotential have prompted relevant researchers to pursue continuous improvements.

A layer of “scaffold” structure is constructed on the surface of the lithium metal anode using principles and methods via nanointerface engineering. This structure exhibits favorable chemical stability and mechanical strength, thereby inhibiting the growth of lithium dendrites during the charging and discharging processes.^67,68^ The utilization of 3D printing of lithium-loaded “scaffolds” enables the personalization of structural designs in accordance with the lithium deposition characteristics and lithium dendrite growth laws, thereby facilitating the stabilization of electrode electrochemical properties. Cao et al. has demonstrated that cellulose nanofibers (CNFs), which are abundant in nature, can be used as an ideal 3D printing ink due to their extensive storage in aqueous solution.^69^ The authors successfully realized the microstructure design of lithium metal by 3D printing, utilizing the CNFs solution as the printing ink. The electrodes produced by this method have a porous internal structure with high ion permeability, which effectively reduces the local current density of the lithium anode and inhibits the formation of dendrites due to uneven lithium deposition and exfoliation. Lyu et al. have further expanded on their previous 3D printing research with a new printing material, which is zinc metal–organic framework (Zn-MOF).^70^ As shown in Figure 2(a), this approach makes effective use of the process of volatilization of zinc metal at high temperatures to design and construct a class of nitrogen-doped carbon framework electrodes. The 3D-printed N-doped carbon framework (3DP-NC) incorporates the advantages of electrode structures, including a graded porous structure, high specific surface area, and nitrogen-doped carbon. The application of this structure in lithium metal electrodes not only facilitates the uniform deposition of a large amount of lithium metal, thus substantially increasing the area-specific capacity (up to 30 mAh·cm^–2^), but also effectively inhibits the growth of lithium dendrites and improves safety, as well as reducing the local current density, which leads to an improvement in the multiplicative performance. Ni et al. prepared porous graphene oxide (GO) structures by stamping GO films with resin templates produced by 3D printing. Subsequently, they obtained porous GO/Li composite electrodes by combining the porous GO films with molten lithium (Figure 2(b)).^71^ The porous structure of the GO substrate provides a transport channel that moderates and uniformly distributes lithium-ion fluxes, thereby avoiding the formation of lithium dendrites to a certain degree. Furthermore, the porous structure is employed in the prevention of lithium dendrites in the anode. Yang’s group employed 3D-printing GO frameworks as lithium–metal framework to regulate the plating behavior of lithium-ions at the interface.^72^ As shown in Figure 2(c), the utilization of 3D printing GO frameworks has been demonstrated to effectively reduce the local current density and provide a larger space for lithium to buffer the volumetric changes. The 3D-GO@Li anode can achieve dendrite-free lithium deposition/peeling with overpotentials as small as 9 mV and has demonstrated a performance of 1600 h at 1 mA·cm^–2^ with a long-term cycling stability.

**Figure 2:**
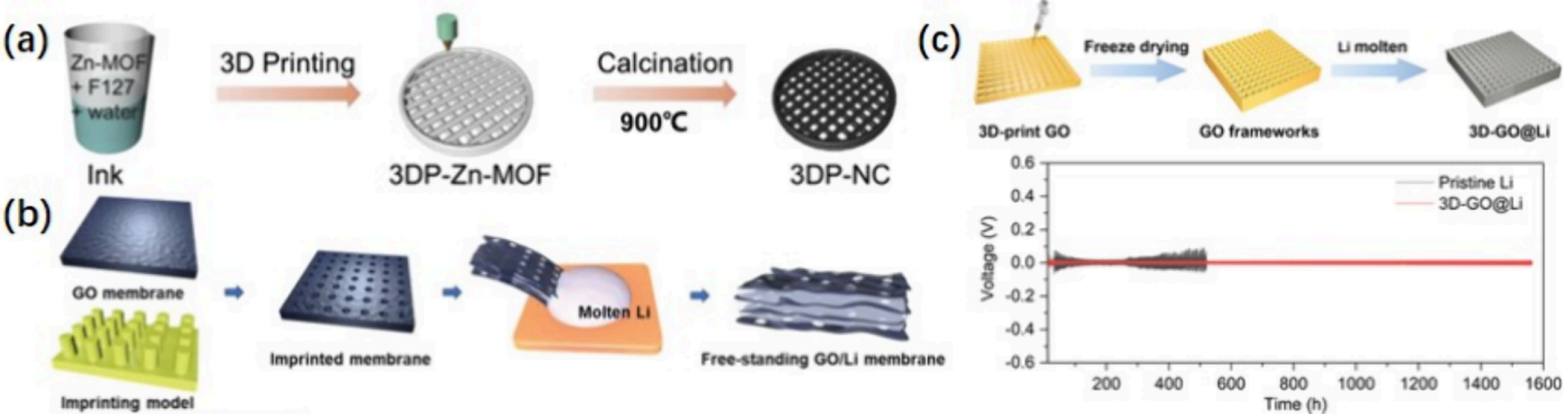
(a) Schematic diagram of the 3DP-NC preparation process;^70^ (b) Schematic diagram of the fabrication process of holey GO/Li electrodes;^71^ (c) Schematic diagram of the fabrication process of 3D-GO@Li negative electrodes and the voltage distribution of symmetric cells with pristine Li and 3D-GO@Li at 1 mA·cm^–2^.^72^ Figure 2 (a): Reprinted with permission from Lyu, Z.; Lim, G. J.; Guo, R.; Pan, Z.; Zhang, X.; Zhang, H.; He, Z.; Adams, S.; Chen, W.; Ding, J. J. E. S. M., 3D-printed electrodes for lithium metal batteries with high areal capacity and high-rate capability. 2020, 24, 336–342. Copyright 2020 Elsevier. Figure 2 (b): Reprinted with permission from Ni, S.; Sheng, J.; Zhang, C.; Wu, X.; Yang, C.; Pei, S.; Gao, R.; Liu, W.; Qiu, L.; Zhou, G. J. A. F. M., Dendrite-free lithium deposition and stripping regulated by aligned microchannels for stable lithium metal batteries. 2022, 32 (21), 2200682. Copyright 2022 John Wiley and Sons. Figure 2 (c): Reprinted with permission from Yang, Y.; Ai, L.; Yu, S.; He, J.; Xu, T.; Chen, D.; Shen, L. J. A. A. E. M., 3D-printed porous go framework enabling dendrite-free lithium–metal anodes. 2022, 5 (12), 15666–15672. Copyright 2022 American Chemical Society.

#### 3.1.2 Carbon-Based

The true underlying motivation for the advent of commercial lithium-ion batteries can be attributed to the integration of carbon materials within lithium-ion battery technology.^73^ Carbon materials can be broadly classified into two main categories, containing graphitized carbon and amorphous carbon. Graphitized carbon has been the subject of more extensive research and has gained wider recognition.^74^ Graphitized carbon can be divided into natural, man-made and composite graphite.^75^ Amorphous carbon is composed of two distinct forms which is hard carbon and soft carbon. Hard carbon encompasses petroleum coke and carbon fiber, whereas soft carbon includes resin carbon, polymer pyrolysis carbon, biomass pyrolysis carbon, and other similar forms.^76^ Graphite is an allotropic form of carbon, defined by a layered structure in which carbon atoms within a layer are connected to their three neighboring carbon atoms by sp^2^ hybridization, forming planar σ-bonds. Atoms that are not involved in this process form a π-conjugated on both sides of the plane, with the layers connected by van der Waals forces.^77–79^ The formation of graphite flakes is attributed to sp^2^ hybridization in graphite, whereby the flakes are typically stacked in the form of ABAB or ABCABC.^80^ The carbon currently employed in large-scale commercialization is graphite, which has a theoretical specific capacity of 372 mAh·g^–1^.^81^ Indeed, graphite is composed of two distinct types of crystals including hexagonal and rhombic. These crystals coexist in graphite in varying proportions.^82^ In general, rhombic crystals are theorized to possess a higher specific capacity than hexagonal crystal phase. Accordingly, modifying the proportion of the two structures can enhance the specific capacity of graphite. The charge and discharge processes of carbon materials are susceptible to complications, including the tendency to co-embed in organic solvents and the formation of unstable SEI films.^83,84^

In order to mitigate the incidence of these issues in the charging and discharging process of carbon materials employed as negative electrode materials, it is common practice to modify these carbon materials.^85^ A multitude of carbon materials exists, and the modification techniques are evaluated in accordance with the specific materials. Among the methods, the utilization of 3D printing technology enables the design and control of specific structures, thereby greatly increasing the freedom of battery geometry design. In turn, this allows for the use of anode materials with enhanced performance.^86^ Traditional manufacture of carbon electrodes involves the use of glassy carbon or carbon fiber with the electrodes themselves being limited in their shapes. The utilization of 3D printing technology in the fabrication of carbon electrodes represents a pivotal aspect of the electrode’s design. As described in Figure 3, Jun’s group prepared a graphite foam (HGF) with high mechanical properties and a periodic porous structure using two modern industrial technologies which is light-cured 3D printing and chemical vapor deposition (CVD).^87^ The material exhibits a periodic porous structure and favorable mechanical properties. Ultimately, the objective of achieving high mechanical strength and ultrahigh active material loading for electrodes is successfully attained. High-performance comb-shaped natural graphite (NG) electrodes are constructed by low-temperature DIW 3D printing by Xu et al.^88^ The properties of the printable NG ink and the performance of the 3D NG electrodes with thicknesses of 347.3 μm, 581.7 and 786.7 μm are also investigated. The electrodes, which have been designed using this method, exhibit a 3D porous microstructure with a porosity level reaching as high as 54.84%. In comparison with strip-cast electrodes of an equivalent thickness, the capacity decay is significantly diminished with an increasing discharge rate when employing this specific 3D electrode structure, thus illustrating the prospective utility of 3D-printed NG electrodes in high-performance lithium-ion batteries. The Jiang’s group prepared graphene oxide electrodes comprising independent rod-like structures through 3D printing.^89^ The method does not rely on the use of molds, thereby reducing the generation of waste and the associated costs. The printed graphene oxide-based electrodes with 3D porous structure can effectively promote the diffusion and transfer of electrons and ions, thereby enhancing the conductivity and electrochemical performance of the battery.

**Figure 3:**
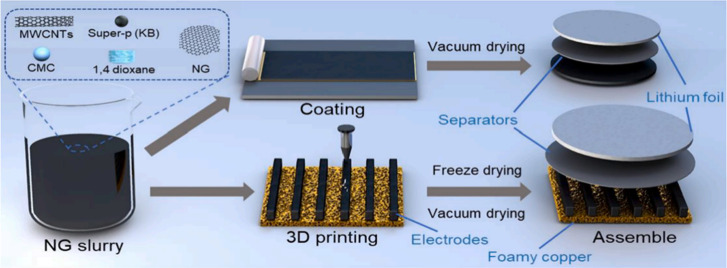
Schematic of the 3D printed NG electrode.^88^ Reprinted with permission from Xu, K.; Zhao, N.; Li, Y.; Wang, P.; Liu, Z.; Chen, Z.; Shen, J.; Liu, C. J. E. C., 3D printing of ultrathick natural graphite anodes for high-performance interdigitated three-dimensional lithium-ion batteries. 2022, 139, 107312. Copyright 2022 Elsevier.

#### 3.1.3 Silicon

Silicon is regarded as the most promising anode material for lithium-ion batteries due to its high theoretical specific capacity, low operating potential, and the wide range of sources from which it has the potential to be sourced.^90,91^ In 1976, Rockwell International identified the benefits of Si as an anode material and employed it for the first time in lithium-ion batteries.^92–94^ Silicon anode belongs to alloy-type anode materials, whose lithium ions are stored by combining with silicon to form a silicon–lithium alloy.^95^ Nevertheless, the material displays limitations in electrical conductivity and exhibits considerable volume effects during its application. Such processes not only result in the destruction of the electrode’s conductive network, leading to the loss of active substances, but also have a detrimental impact on the cycling performance of silicon anode materials.^96–98^ The key to achieve the electrochemical stability of silicon anodes is to mitigate volume change and improve charge carrier transport capacity.^99^ The deformation of special shaped silicon crystal materials, such as honeycomb and dendritic silicon materials, can be leveraged to absorb the volume change during charging and discharging, thereby enhancing the cycling performance of silicon materials.^100,101^ Nevertheless, the electrode compaction density is relatively low, and the process is complex and challenging to prepare. To enhance the electrochemical functionality of silicon electrodes, porous structures are typically incorporated during the structural formula design process. This approach mitigates the volume expansion of silicon and minimizes the occurrence of side reactions induced by the volume effect.^102^

In a recent publication, Mu et al. put forth a novel approach to developing a self-supported silicon-graphene (3D-Si/G) anode with an ultrahigh surface capacity, as described in Figure 4. This method involves manipulating the electrode structure through the use of 3D printing technology.^103^ The 3D-Si/G electrode with a circular grid pattern allows for the precise construction and adjustment of electrode thickness as well as the spacing of each electrode. The unique configuration of this structure allows for the accommodation of volume expansion in silicon anode, thereby enhancing the structural stability and integrity of the electrode. Furthermore, it facilitates the transport of lithium ions in thick electrodes. Beydaghi et al. have prepared silicon-based electrodes and employed them as anodes for lithium-ion batteries via a straightforward and cost-efficient FDM approach.^104^ The FDM method is based on polylactic acid as the principal polymer matrix, and a mixture of conductive carbon black and graphene flakes synthesized from polypyrrole as a precursor is used as a conductive additive to the conductive material, with the objective of optimizing electrochemical performance. This method permits the construction of a continuous conductive network and the control of the electrode microstructure at the nanoscale, thereby enabling the precise regulation and controllable construction of the microstructure of the silicon anode. The lithium-ion battery with this electrode as the anode exhibits a specific capacity of 345 mAh·g^–1^ at a current density of 20 mA·g^–1^, with a capacity retention rate of 96% after 350 cycles. In general, the porous structures manufactured by 3D printing technology provide free space for the volume change of silicon electrode during charging and discharging processes, which is benefit for improving the cycle life of electrode materials.

**Figure 4:**
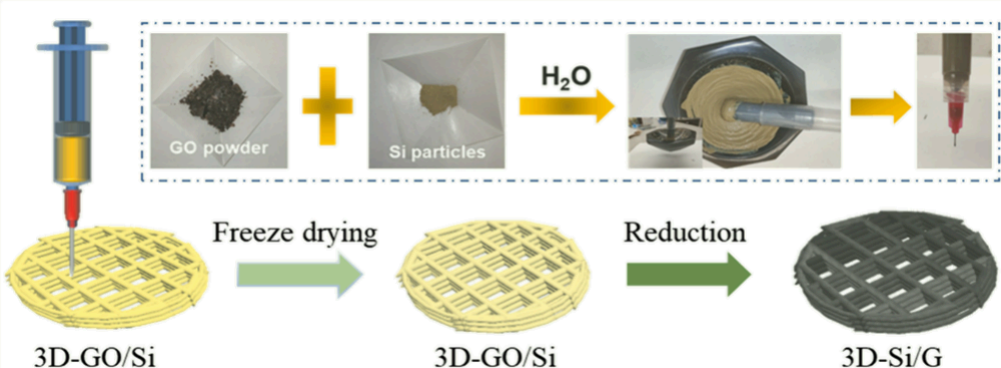
Schematic diagram of the preparation process of 3D-Si/G electrode (inset shows the preparation process of GO-Si ink).^103^ Reprinted with permission from Mu, T.; Xiang, L.; Wan, X.; Lou, S.; Du, C.; Zuo, P.; Yin, G. J. E. S. M., Ultrahigh areal capacity silicon anodes realized via manipulating electrode structure. 2022, 53, 958–968. Copyright 2022 Elsevier.

### 3.2 Cathode Materials

#### 3.2.1 LiCoO_2_ (LCO)

Lithium cobalt oxide (LiCoO_2_) is one of the most commonly employed cathode materials in lithium-ion batteries, primarily due to its elevated specific energy (150–200 Wh/kg) and high operating voltage (3.7 V).^105^ The crystal structure of LCO is hexagonal, belonging to the *R*3̅*m* space group. In the crystal structure, the cobalt ions (Co^3+^) are situated in the coordinating positions of the octahedron, while the lithium ions (Li^+^) are located within the octahedral voids. This structure enables high ion mobility rates and superior charge transport performance, thereby conferring upon LCO excellent cycling stability and a prolonged service life.^106^ However, LCO is expensive and prone to thermal runaway reactions at high temperatures, leading to safety issues.^107^ Xu et al. have prepared a crossover lithium-ion battery comprising a comb-shaped three-dimensional high-voltage LiCoO_2_ (HV-LCO) cathode and a comb-shaped three-dimensional natural graphite anode through the use of 3D printing technology.^108^ Furthermore, printable HV-LCO inks with the requisite rheological properties are developed for 3D printing. The electrochemical performance has evaluated through the examination of the HV-LCO half-cell with lithium foil acting as the counter electrode, as well as the full-cell with NG anode serving as the counter electrode. The results demonstrate that the crossover full cells fabricated by 3D printing exhibit high specific capacity and stable cycling performance. The full cell with an electrode thickness of 882 μm exhibits a high capacity of 5.88 mAh·cm^–2^@0.1 C, energy density of 41.4 J·cm^–2^, and power density of 41.0 mW·cm^–2^@1.0 C, which is approximately 10 times that of the conventional thin cell. Yee et al. have fabricated LCO macrostructures by first synthesizing homogeneous lithium and cobalt nitrate aqueous photo resins, which are then used in conjunction with 3D printing method to obtain Li^+^ and Co^3+^ containing hydrogels.^109^ The 3D hydrogels are subjected to calcination in order to obtain micropore self-supporting LCO structures with a resolution of approximately 100 μm. These freestanding, binder-conductive additive-free LCO structures are integrated as cathodes into lithium-ion batteries and exhibited 76% electrochemical capacity retention over 100 cycles at 0.1C, demonstrating their suitability in lithium-ion batteries. The simplicity of this method for fabricating LCO structures can be extended to other materials by modifying the properties of the 3D printing solution, thereby providing a general method for fabricating multicomponent metal oxides with complex 3D structures. As shown in Figure 5, Fu et al. have reported a strategy for the design of 3D-printed electrode structures with ordered channels for the construction of high-voltage LCO ultrathick cathodes. 3D printed LCO thick electrodes are a great option for batteries due to their lithium-ion diffusion coefficient (3.8 times higher). The 3D-printed LCO |Li cell (29 mg cm^–^^2^) delivers a high areal capacity (5.16 mAh cm^–^^2^) and 89% capacity retention after 200 cycles (3 mA cm^–^^2^).^110^

**Figure 5:**
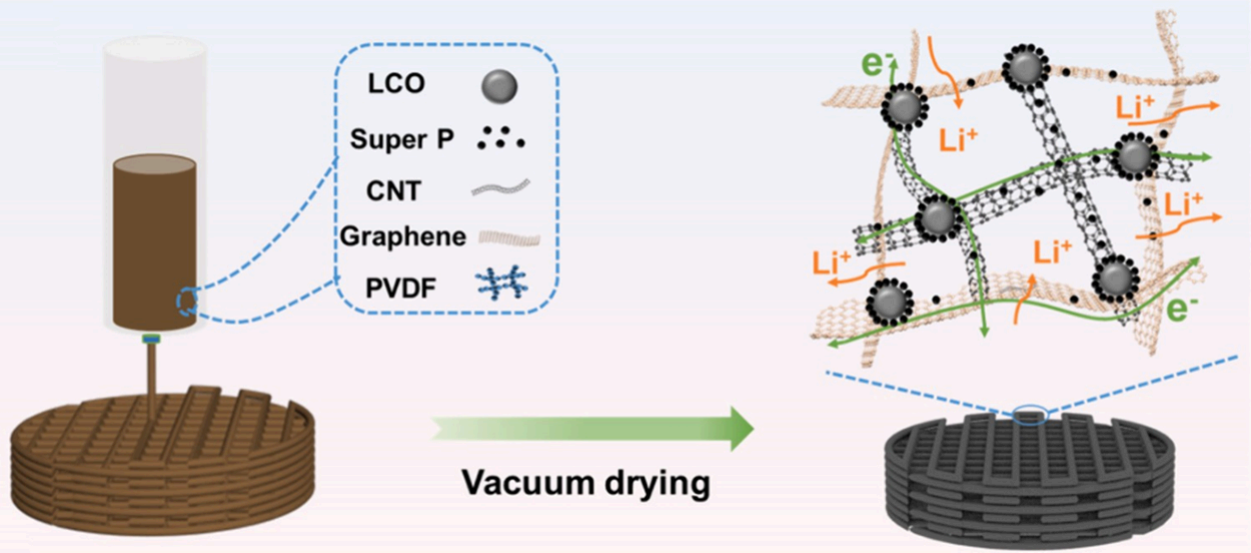
Schematic diagram illustrating the fabrication of 3D-printed LCO electrodes and composition of LCO ink.^110^ Reprinted with permission from Fu, Y.; Zhang, A.; Ma, J.; Bi, Z.; Guo, Z.; Ma, Y.; Liao, S.; Qu, J.; Li, C.; Wu, Z.-S., Ultrathick LiCoO2 cathodes with low tortuosity and accelerated kinetics enable high areal capacity and long-life customable batteries. Energy Storage Materials 2025, 78. Copyright 2025 Elsevier.

#### 3.2.2 LiFePO_4_ (LFP)

Lithium iron phosphate (LiFePO_4_), frequently abbreviated as LFP, offers a multitude of advantages, including high energy density, extended cycle life, minimal self-discharge, and commendable safety characteristics. It is a vital cathode material for lithium-ion batteries, with a pervasive presence in electric vehicles, energy storage systems, and portable devices.^111^ The crystal structure of LFP belongs to the orthorhombic crystalline system, with a space group of *Pnma*. In the crystal structure, iron ions (Fe^2+^) are in the coordination positions of the octahedron, lithium ions (Li^+^) are in the octahedral voids, and phosphate ions (PO_4_^3–^) connect Fe^2+^ and Li^+^. In comparison to alternative cathode materials, LFP exhibits superior power density and enhanced thermal stability, which collectively contribute to an extended cycle life and enhanced safety performance. Furthermore, LFP has lower raw material costs and is relatively more environmentally friendly, which is one of the reasons for its widespread use in Li-ion batteries.^112^ While LFP possesses lots of advantageous characteristics, it is nevertheless subject to certain constraints. First, the low voltage plateau (∼3.4 V) necessitates the utilization of a greater quantity of material to achieve an equivalent energy density, which consequently results in an increase in the dimensions and weight of the battery. Furthermore, LFP exhibits a reduced rate of ion migration, which consequently results in a diminished charge/discharge rate.^113^ To address these challenges, researchers are developing enhanced lithium ferrous phosphate materials through techniques such as surface modification, alloying, and nanosizing. These methods aim to enhance the material’s conductivity and charge/discharge rate. Additionally, researchers are exploring alternative materials to further optimize the performance of lithium-ion batteries.

Cong et al. have employed 3D printing technology to prepare LFP porous electrodes­(Figure 6(a)).^114^ The results demonstrate that the apparent viscosity of the printing ink at a constant shear rate increased in proportion to the LFP content, rising from 20 wt % to 60 wt %. Similarly, the yield stress increases from 203 to 1187 Pa. Tests of rheological and printability slurry performance has revealed that the ink with 40 wt % LFP has exhibited excellent properties. Its porous skeleton has reduced the ion and electron transport distance, enhanced the efficiency of the electrode reaction, and optimized the multiplicity performance and cycling stability. Following 3D printing technology, the LFP electrode with an active material loading of 15.9 mg·cm^–1^ exhibits a specific capacity of 121.7 mAh·g^–1^, coulombic efficiency exceeding 99.7%, and an energy density of 350 Wh·kg^–1^ after 200 cycles at 0.5 C. Bao et al. have demonstrated that 3D printed LFP/LTO electrodes achieve 92% (LFP) and 88% (LTO) capacity retention during 100 discharge/charge cycles­(Figure 6(b)).^115^ It is of greater significance that the soft pack battery with LFP/LTO as electrodes exhibits a high discharge specific capacity of approximately 120 mAh·g^–1^ at 0.3 C, coupled with favorable mechanical properties. This offers a novel concept for the conceptualization and construction of stretchable energy storage systems, with the potential to facilitate the development of future wearable and stretchable electronic products.

**Figure 6:**
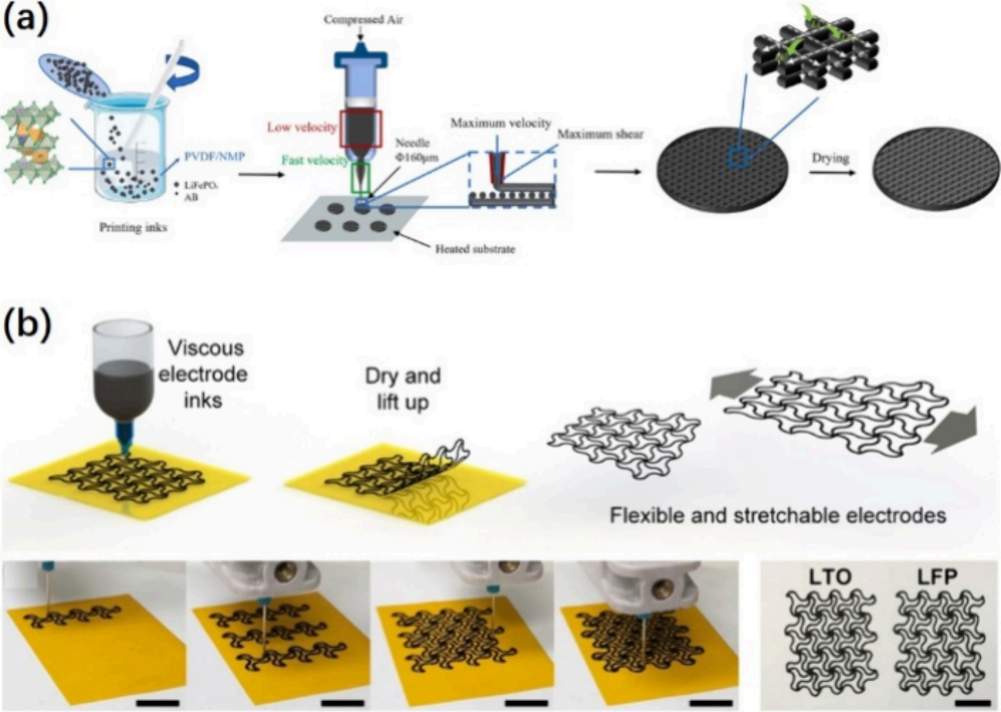
(a) Fabrication process for the preparation of comb LiFePO_4_, SiO@C/graphite 3D electrodes, and staggered full cells by low-temperature direct-write 3D printing;^114^ (b) Suitably patterned stretchable electrodes fabricated by 3D printing: fabrication process, optical photographs of different steps in the printing process, and fabricated stretchable LTO and LFP electrodes.^115^ Figure 6 (a): Reprinted with permission from Cong, M.; Du, Y.; Liu, Y.; Xu, J.; Zhao, K.; Lian, F.; Lin, T.; Shao, H. J. C. I., Preparation of lithium iron phosphate battery by 3D printing. 2024. Copyright 2024 Elsevier. Figure 6­(b): Reprinted with permission from Bao, Y.; Liu, Y.; Kuang, Y.; Fang, D.; Li, T. J. E. S. M., 3D-printed highly deformable electrodes for flexible lithium ion batteries. 2020, 33, 55–61. Copyright 2020 Elsevier.

### 3.3 Solid State Electrolyte

Currently, electrolyte is a vital electrically conductive medium used in lithium-ion batteries. It is typically a mixture of lithium salts in an organic solvent. The lithium salt may be any of various compounds, including lithium fluoride (such as LiPF_6_, LiBF_4_), lithium sulfonate, lithium phosphate, and so forth.^116–119^ The organic solvent may be any of a number of different chemical compounds, including carbonate, polycarbonate, ester, *etc.*. The primary function of the electrolyte in a lithium-ion batteries is to facilitate the transport of lithium ions and to ensure the battery’s optimal functioning.^120^ Concurrently, the electrolyte assumes a pivotal function in regulating the internal temperature of the battery, preventing short-circuiting of the battery, and providing a stable voltage. However, liquid electrolytes have a low flash point and narrow voltage window, which affects the safety of the battery and further increases in energy density. The replacement of the liquid electrolyte with a solid electrolyte, which is nonflammable and exhibits enhanced compatibility with the lithium metal anode, enables the development of a novel battery configuration, namely the solid state battery. This innovation promises to enhance the energy density and safety performance of the battery. A solid state electrolyte is usual type of inorganic or organic solid material employed in lithium-ion batteries for the transmission of lithium ions, offering enhanced safety and stability. The use of solid state electrolytes has the potential to impede the occurrence of thermal runaway reactions in lithium-ion batteries, thereby enhancing the overall safety of the battery^121,122^ Furthermore, they can facilitate higher ionic conductivity, which enhances battery performance and prolongs the battery cycle. Solid state electrolytes may be composed of inorganic materials, including oxides, sulfides, or phosphates, or organic polymers.^123^ The current challenges of solid state electrolytes include the improvement of ionic conductivity, the enhancement of interfacial stability, and the reduction of preparation costs.^124^

Gambe et al. has successfully developed a 3D printable gel electrolyte that can be extrusion printed at room temperature and main whole structure even after stacking multiple layers with good integrity (Figure 7). Moreover, the electrolyte gel cures by UV irradiation while maintaining its structural integrity and high lithium ion conductivity, which results in fully 3D printed quasi-solid-state lithium-ion batteries.^125^ The method presented here shows promise for the simple fabrication of thermally unstable electronic devices, such as flexible devices and biocompatible micro sensors. The fabrication of 3D printing quasi-solid PLA–PEO electrolytes injected with LiTFSI ionic liquids is achieved by Vinegrad et al. through the use of fused filament fabrication of printed poly­(propylene glycol)-poly­(ethylene oxide) hybrid membranes.^126^ A comparison of the ionic conductivity properties of ionic liquid-plasticized quasi-solid 3D printing electrolytes of pristine PLA and PLA–PEO printed blends reveals that pure PLA exhibits poor lithium-ion conductivity. Conversely, an increase in the number of carriers leads to an increase in bulk conductivity. The cycling of a symmetrical lithium battery with a printed plasticized electrolyte for 140 h has demonstrated that the battery exhibits a stable profile with relatively low polarization and no indication of lithium dendrite growth. The elevated charge/discharge overpotential is attributed to the considerable impedance observed in the printed cathode cell, which employs PLA polymer with limited conductivity as a binder for the LFP. Furthermore, the minimal quantity of ionic liquid (IL) has incorporated into the printed plasticized electrolyte (PPE) is inadequate to facilitate a low-resistance printed electrode/PPE interface contact. Ultimately, the current density has increased by a factor of 4, thereby achieving reversible cyclability of the battery. Zhang et al. have proposed 3D printing with zwitter molecule modification to improve lithium–metal battery performance. Aspartate acid carboxyl groups donate electrons, changing polyvinylidene fluoride (PVDF) to enhance Li^+^ transport and anion immobilization on polymer chains. The amphoteric groups of Asp promote dissociation of lithium salts and desolvation of lithium ions with DMF, stabilizing Li_3_N/LiF-enriched interphases. This creates an SPE with high ionic conductivity (1.2 × 10^–4^ S·cm^–1^), large transfer number (0.68) and tensile strength (∼110 MPa).^127^

**Figure 7:**
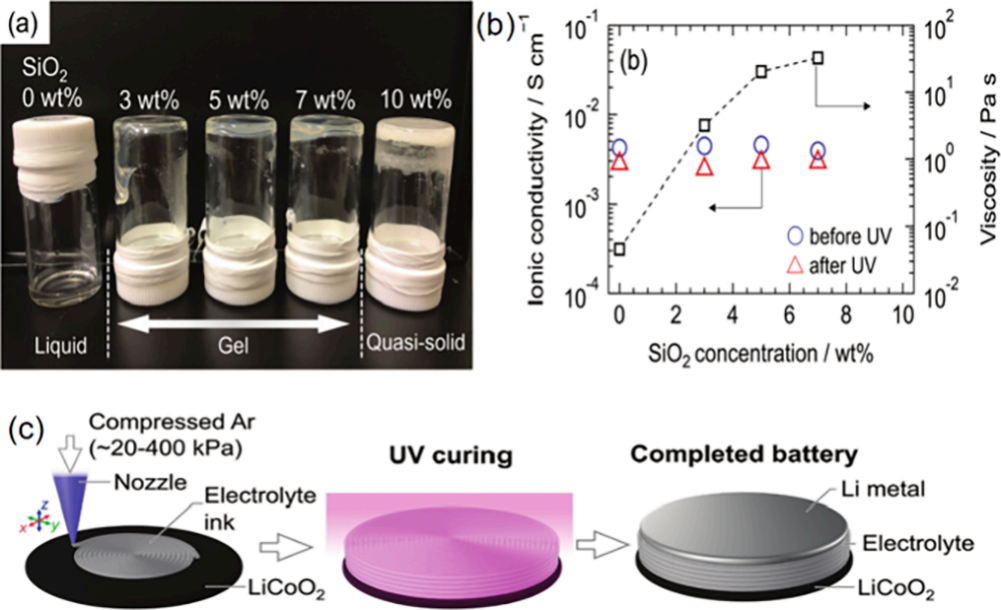
(a) Photograph of an electrolyte ink consisting of an ionic liquid, UV-cured polymer, and SiO_2_ nanoparticles; (b) SiO_2_ concentration dependence of ionic conductivity and viscosity; (c) Schematic of the geometry of a 3D printed, UV-cured, and completed quasi-solid lithium-ion batteries of a gel electrolyte ink.^125^ Reprinted with permission from Gambe, Y.; Kobayashi, H.; Iwase, K.; Stauss, S.; Honma, I. J. D. T., A photocurable gel electrolyte ink for 3D-printable quasi-solid-state lithium-ion batteries. 2021, 50 (45), 16504–16508. Copyright 2021 Royal Society of Chemistry.

### 3.4 Performance Comparison

The electrochemical properties comparation for electrode/electrolyte fabricated by traditional method and 3D printing are shown in Table 2. The traditional electrode manufacturing relies on coating technology, which is difficult to accurately control the microstructure, resulting in the agglomeration of active particles and the ion transport bottleneck. Through structural innovation and process innovation, 3D printing not only makes up for these defects, but also provides a new path for high energy density and high safety batteries. The specific capacity, life span and mass loading of cathode and anode made by 3D printing show obvious improvements compared with traditional method. In addition, the 3D-printed electrolytes show better electrical conductivity.

**Table 2:** Electrochemical Properties Comparation for Electrode/Electrolyte Fabricated by Traditional Method and 3D Printing

		materials	**specific capacity** mAh/g	**life span** th	**thickness** μm	ref
cathode	traditional method	LiFePO_4_	68	30	50	^128^
		LiCoO_2_	5.2	100	130	^129^
		LiMn_2_O_4_	∼110	21	160	^130^
	3D printing	LiFePO_4_	82	30	90	^128^
		LiCoO_2_	137.4	40	∼195	^129^
		LiMn_2_O_4_	∼117	21	370	^130^
**anode**	**traditional method**	C	∼285	30	60	^131^
		Si	245	200		^132^
		Sn	468	30		^133^
		Li_4_Ti_5_O_12_	<10		∼500	^134^
		Li	∼80	3000		^135^
	**3D printing**	C	∼326	30	250	^131^
		Si	345	350	200	^136^
		Sn	∼200	∼50	2.3	^137^
		Li_4_Ti_5_O_12_	∼145	100	1085	^134^
		Li	∼2346	300	200	^138^
			**conductivity** S/cm			
**electrolyte**	**traditional method**	PEO	3.92 × 10^–^^4^ (25 °C)			^139^
		GPE	3.6 ± 0.7 × 10 ^–3^ (25 °C)			^140^
		LITFSI	3.92 × 10^–^^4^ (25 °C)			^139^
	**3D printing**	PEO	2.18 × 10^–^^3^ (90 °C)			^139^
		GPE	3.3 ± 0.6 × 10^–^^3^ (25 °C)			^140^
		LITFSI	2.18 × 10^–^^3^ (90 °C)			^139^

## 4 Prospects

In recent years, 3D printing technology has been extensively utilized in the domain of electrochemical energy storage, garnering the interest of numerous researchers due to the highly customized and on-demand capabilities of additive manufacturing. It is evident that 3D printing technology offers a multitude of advantages in the domain of lithium-ion battery applications. In comparison with conventional fabrication methodologies, 3D printing offers distinctive advantages in the domain of electrode/electrolyte design. Nevertheless, it is imperative to address critical challenges to fully realize its potential. A notable benefit of 3D printing is its ability to overcome the constraints imposed by two-dimensional coating methodologies. Conventional electrodes, constrained by planar geometries, typically exhibit thicknesses below 200 μm and porosities ranging from 30% to 40% to avoid delamination. Conversely, 3D printing architectures facilitate precise regulation of pore distribution, material loading, and geometric complexity. In addition to its structural complexity, 3D printing also facilitates the customization of electrode geometry. However, it should be noted that such advancements are not without their drawbacks. Electrodes manufactured using the 3D printing frequently necessitate the use of inks that are tailored for the specific requirements of the process, and the implementation of postprocessing steps such as thermal annealing or UV curing. These steps can introduce complexity to the manufacturing workflow. Second, traditional slurry-coating methods result in a considerable amount of material waste. This is due to the uneven distribution of binder and the inclusion of nonactive components, such as conductive additives. The mass percentage of these additives in the total electrode mass typically ranges from 15 to 30%. The process of 3D printing, however, circumvents these inefficiencies by depositing materials only where they are required, thereby achieving a near-net-shape fabrication. Nevertheless, challenges persist in the formulation of inks. A significant number of 3D printing electrodes continue to utilize thermoplastic binders (for example, PLA and ABS) which exhibit insufficient ionic or electronic conductivity. In order to address this issue, researchers have developed composite inks combining conductive polymers (for example, PEDOT:PSS) with nanostructured active materials. The field of safety represents a further critical domain in which 3D printing offers distinct advantages. Solid state electrolytes (SSEs) printed via stereolithography have been shown to exhibit ionic conductivities of 3.3 × 10^–^^3^ S·cm^–1^ at 25 °C, which rivals the conductivity of liquid electrolytes while eliminating the risk of leakage.

In summary, 3D printing technology has the potential to revolutionize the manufacturing of lithium-ion batteries. However, several challenges must be overcome before this can be realized. It is evident that further research and improvements are necessary to fully realize the benefits of 3D printing technology in this field. Nonetheless, as technology advances, it is anticipated that these challenges will be progressively addressed, thereby opening a wider range of possibilities to produce lithium-ion batteries.
